# A study on the aromatic conjugation pathways and the ring currents of bridged [18]annulenes†

**DOI:** 10.1039/c9ra04193a

**Published:** 2019-08-14

**Authors:** Qimanguli Tuoheti, Ablikim Kerim

**Affiliations:** The College of Chemistry and Chemical Engineering, Xinjiang University Urumqi 830046 China ablikim.kerim@163.com

## Abstract

In this article, the topological resonance energy (TRE) method was used to investigate the global aromaticity of a set of [18]annulene-derived compounds which were obtained by replacing either two, four, or all six of the inner hydrogen atoms of [18]annulene with bridges (oxygen, imino-, sulfur, or combinations of the three). Our TRE results show that all the mono-bridged, di-bridged, and tri-bridged [18]annulenes are globally aromatic systems with positive TREs and show relatively larger aromaticity in comparison with the [18]annulene. The aromaticity of each compound was explained using the topological charge stabilization (TCS) rule. The bond resonance energy (BRE) and circuit resonance energy (CRE) methods were used to evaluate local aromaticity. Our BRE and CRE results show that incorporation of five-membered heterocyclic rings changes the main conjugation pathway of the bridged [18]-annulenes. The local aromaticities arising from the five-membered heterocyclic rings (6π) contribute strongly to global aromaticity. However, the ring current (RC), which arises from the 18π annulene-like pathway structures, is the primary determinant of the magnetic properties of the molecule. Our RC results are in good agreement with available ^1^H-NMR chemical shift data.

## Abstract 2

The topological resonance energy method was used to investigate the global aromaticity of a set of [18]annulene-derived compounds obtained by replacing two, four, or all six of the inner hydrogen atoms with bridges (oxygen, imino-, sulfur, or combinations of the three).

## Graphical abstract

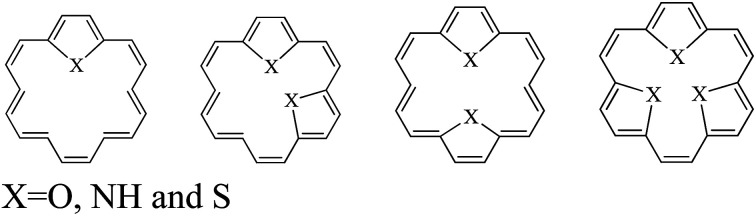

## 1. Introduction

Annulenes are monocyclic conjugated polyenes with alternating single and double bonds. The general formula of this family can be represented as (C_2_H_2_)_*n*_, in which *n* represents the number of carbon atoms in the ring. The chemistry of annulenes has long been a subject of great interest to both synthetic and theoretical chemists.^1,2^ The [18]annulene has been regarded as a fundamental structure in annulene chemistry and has been extensively used as a model system for the study of aromaticity. According to Hückel's rule of aromaticity, because the [18]annulene contains (4*n* + 2) π-electrons, it must be aromatic, and its ^l^H-NMR data^3^ confirms the existence of strong diatropic ring currents (RC). Six interior hydrogen atoms are present in [18]annulene. Pairs of interior hydrogen atoms in the [18]annulenes are replaced by either one, two, or three oxygen atoms, imino atoms, or sulfur atoms, or by any combination of these oxygen-, imino-, or sulfur atom groups. Since Badger *et al.*^4^ first reported the synthesis of [18]annulene trisulphide, bridged [18]annulenes have been the subject of much research in both theoretical and experimental organic chemistry. These compounds belong to a family of polycyclic aromatic compounds. Because the structure of bridged [18]annulenes contains one, two, or three five-membered heterocyclic rings fused to an [18]annulene core, they are similar to [18]annulene in structure. Because bridged [18]annulenes contain an increased number of π-electrons and one, two, or three five-membered heterocyclic rings within the molecule, an intriguing new cycle is produced, which results in changes in the aromatic pathways of the molecule. The magnetic circular dichroism spectra and the aromatic stability of some of the bridged [18]annulenes have been reported previously.^5–8^ However, there have been no systematic studies on the aromaticity and the magnetic properties of the mono-bridged, di-bridged, and tri-bridged [18]annulenes.

Aromaticity is one of the important concepts in understanding the chemical properties of annulene-like compounds. There are numerous computational criteria of aromaticity.^9–13^ Topological resonance energy (TRE) is one of the energetic indices of aromaticity based on π-electron delocalization and it has been successfully utilized to explain many chemical phenomena.^14–18^

The determination of the main source of aromaticity is the most important task in the study of polycyclic compounds and has attracted much attention.^19–22^ In these compounds, every pathway contributes to a greater or lesser extent, to its global aromaticity. The questions arise as to how the aromaticity of bridged [18]annulenes is different from the aromaticity of [18]annulene and whether or not it is the 18π electrons in the bridged [18]annulenes perimeter that determines the aromaticity of the molecule as a whole. It is very important to discover the source of the global and local aromaticity as well as the magnetic properties of bridged [18]annulene compounds.

In this paper, using the TRE method,^14–18^ we investigate the global aromaticity of [18]annulene before and after the replacement of two, four, and six of its interior hydrogen atoms with heteroatoms X (X = O, and/or NH, and/or S), which produces heteroatom-bridged [18]annulenes. Bond resonance energy (BRE)^23^ and circuit resonance energy (CRE)^24,25^ indices of aromaticity are used to predict local aromaticity. Finally, the RC of these compounds is also discussed.

## 2. Methods of calculation

TRE is related to the cyclic delocalized distribution of π-electrons.^14–18^ Positive and negative TREs indicate aromaticity and antiaromaticity, respectively. % TRE, when normalized with respect to the size of the π-system, is defined as TRE multiplied by 100, with that quantity then divided by the total π binding energy of the polyene reference structure. It can be used to compare the aromaticity of a variety of organic molecules. BRE represents the contribution of a given π-bond to the TRE.^23^ If the BRE exhibits a positive value, this indicates a stabilizing contribution of a given π-bond to the molecule as a whole; if the BRE exhibits a negative value, a destabilizing contribution is indicated. The CRE value can be used to measure the contribution of the resonance energy that each circuit makes to the TRE.^24,25^ Both the BRE and CRE can be used to predict the local aromaticity of polycyclic π-bond systems.^26–31^ The TRE, BRE, and CRE are given in units of |*β*|, where *β* is the standard resonance integral in Hückel theory. RCs are analyzed by means of the Hückel–London model.^32–35^ For the heteroatoms, Van-Catledge's set of Hückel parameters have been used here.^36^ With aromatic compounds, as the positive values of TRE, BRE, and CRE increase, so does the aromaticity of those molecules. All the calculations of TRE, % TRE, BRE, CRE, and RCs are performed within the framework of simple Hückel molecular orbital theory.

## 3. Results and discussion

### 3.1. Global aromaticity

**Fig. 1:**
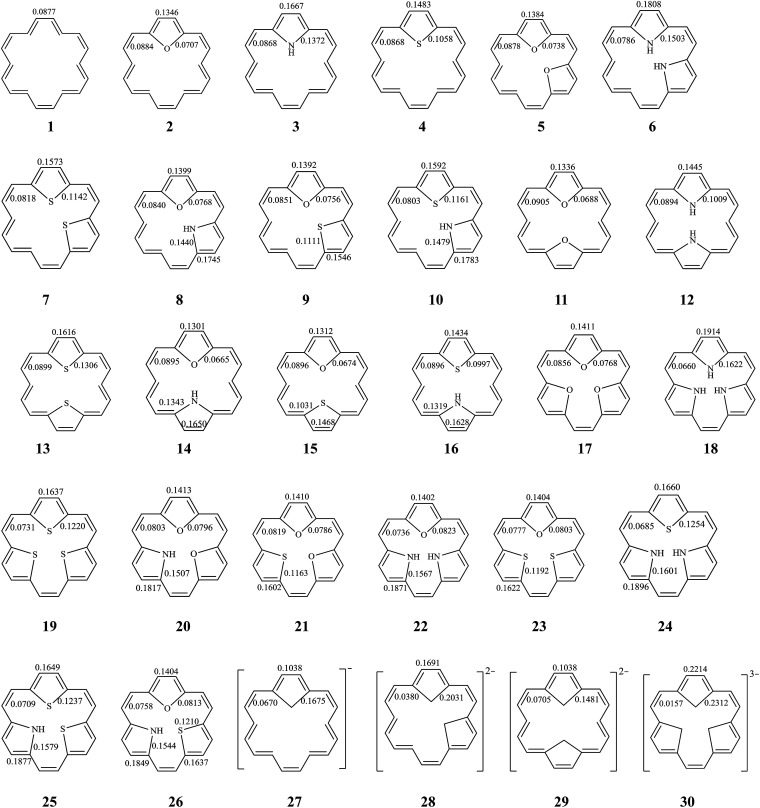
The structure of all possible bridged [18]annulenes (2–26) and their related species. BREs are in units of |*β*| for all π-bonds.

Among the inner six hydrogen atoms in [18]annulene (1), the replacement of two or more of the adjacent hydrogen atoms by differing numbers of, or differing combinations of, X (O, and/or NH, and/or S) atoms (as described above) leads to mono-bridged [18]annulenes (2–4), di-bridged [18]annulenes (5–16), and tri-bridged [18]annulenes (17–26). All possible isomers are shown in Fig. 1. The TRE and % TRE values we calculated are given in Table 1. As shown in Table 1, all the compounds (1–26) are predicted to be aromatic systems with positive TREs. The insertions of a five-membered ring or rings in 1 increase the global aromaticity of 2–26 according to the % TRE values. By comparing the magnitude of the % TRE values, we find first that all the bridged [18]annulenes (2–26) have considerably larger aromaticity than 1, and secondly, that as the number of five-membered rings in the molecule increases, there is an appreciable effect on the delocalization of the electrons, and the aromaticity gradually increases as well. According to the % TRE values, in the isomers where O atom(s) are substituted in, we obtain the following aromatic order: 17 > 5 > 2 > 1. Similar trends also can be found in the other isomers where same-type heteroatoms were substituted in. Among those compounds into which the same type and the same number of heteroatoms have been substituted, the order of aromaticity has been determined as follows: NH group(s) > S atom(s) > O atom(s) > 1. The observed trends in the aromatic properties are related to the differences in the electronegativity while NH, O, and S affect the aromatic ring.^31^ Compounds into which NH group(s) have been substituted have stronger aromaticity than those into which S atoms and O atoms have been substituted, since rings into which NH groups have been substituted display greater delocalization.

**Table 1:** The TRE, % TRE, MRE, and the CRE values of compounds 1–30. The units of TRE, MRE, and CRE are given in |*β*|

Species	TRE	% TRE	MRE	CRE										
				*c*_1_	*c*_2_	*c*_3_	*c*_4_	*c*_5_	*c*_6_	*c*_7_	*c*_8_	*c*_9_	*c*_10_	*c*_11_
1	0.088	0.382	0.071	3.143										
2	0.156	0.570	0.127	0.163	0.521	2.620								
3	0.214	0.811	0.170	0.297	1.062	1.984								
4	0.186	0.725	0.148	0.230	0.867	2.202								
5	0.228	0.717	0.187	0.172	0.172	0.083	0.427	0.427	2.165					
6	0.360	1.212	0.289	0.333	0.333	0.314	0.629	0.629	1.210					
7	0.297	1.049	0.238	0.253	0.253	0.212	0.581	0.581	1.513					
8	0.291	0.945	0.236	0.181	0.314	0.163	0.857	0.317	1.625					
9	0.261	0.869	0.211	0.178	0.244	0.133	0.701	0.355	1.812					
10	0.328	1.131	0.263	0.259	0.326	0.258	0.710	0.515	1.354					
11	0.223	0.699	0.180	0.158	0.158	0.084	0.436	0.436	2.213					
12	0.335	1.126	0.263	0.282	0.282	0.348	0.696	0.696	1.337					
13	0.280	0.990	0.219	0.219	0.219	0.227	0.621	0.621	1.616					
14	0.279	0.905	0.221	0.153	0.289	0.171	0.899	0.332	1.703					
15	0.251	0.836	0.200	0.155	0.223	0.139	0.729	0.369	1.882					
16	0.307	1.060	0.241	0.216	0.285	0.259	0.710	0.516	1.469					
17	0.304	0.838	0.252	0.180	0.180	0.180	0.012	0.066	0.066	0.066	0.346	0.346	0.346	1.773
18	0.526	1.588	0.429	0.369	0.369	0.369	0.076	0.170	0.170	0.170	0.357	0.357	0.357	0.711
19	0.419	1.354	0.342	0.278	0.278	0.278	0.045	0.133	0.133	0.133	0.380	0.380	0.380	1.017
20	0.372	1.056	0.306	0.190	0.190	0.332	0.023	0.127	0.127	0.048	0.684	0.255	0.255	1.319
21	0.340	0.986	0.280	0.186	0.186	0.258	0.019	0.105	0.105	0.054	0.561	0.287	0.287	1.477
22	0.446	1.305	0.365	0.199	0.351	0.351	0.043	0.241	0.091	0.091	0.497	0.497	0.186	0.973
23	0.378	1.157	0.310	0.192	0.268	0.268	0.029	0.164	0.086	0.086	0.462	0.462	0.237	1.228
24	0.489	1.510	0.400	0.289	0.363	0.363	0.064	0.196	0.140	0.140	0.407	0.407	0.294	0.803
25	0.453	1.433	0.371	0.283	0.283	0.356	0.053	0.162	0.162	0.116	0.463	0.335	0.335	0.905
26	0.412	1.231	0.337	0.196	0.274	0.344	0.035	0.199	0.104	0.075	0.564	0.408	0.210	1.094
27	0.183	0.746	0.161	0.308	2.099	0.275								
28	0.397	1.524	0.335	0.448	0.448	0.848	0.253	0.253	0.023					
29	0.314	1.209	0.257	0.296	0.296	1.445	0.394	0.394	0.044					
30	0.679	2.474	0.569	0.550	0.550	0.550	0.154	0.103	0.103	0.103	0.037	0.037	0.037	0.001

**Fig. 2:**
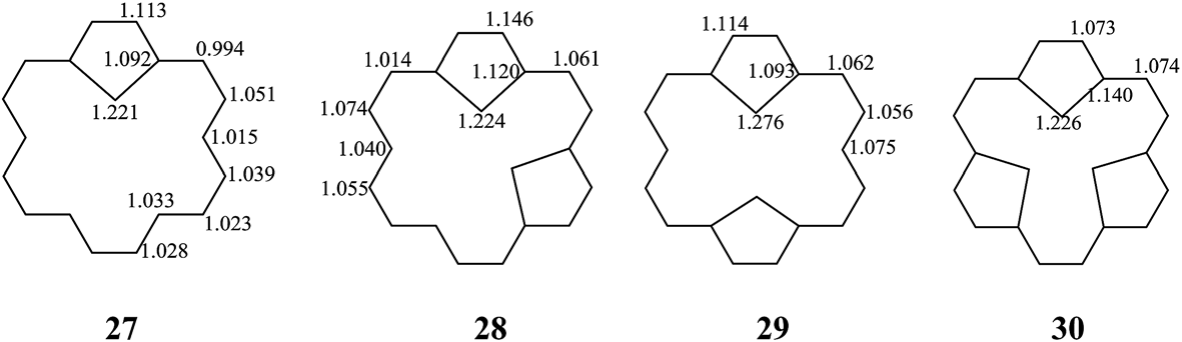
The URFs for mono-bridged (2–4), di-bridged (5–16), and tri-bridged (17–26) [18]annulenes (X = O, NH and S).

The stability of these compounds can be explained in the following manner. According to Gimarc's topological charge stabilization rule (TCS),^37,38^ heteroatomic molecules are stabilized when additional electronegative atoms are placed in those positions which exhibit the highest electron charges in the uniform reference frame (URF) (*i.e.*, the iso-π-electron, isostructural hydrocarbon). The URF for 2–4 is described by URF 27 with 20 electrons. The URF for 5–10 and 11–16 is described by URF 28 and by 29, respectively, with 22 electrons each. The URF for 17–26 is described by URF 30 with 24 electrons. The charge densities of the URF for 27–30 are shown in Fig. 2. As shown in Fig. 2, all the heteroatom substitutions occur at the inner, five-membered ring position which has the highest charge in the corresponding URF. As can be seen, all the compounds (2–26) fully obey the TCS rule and have large TREs and fairly large % TREs. The TREs and % TREs of 27–30 are presented in Table 1. It is noteworthy that the heterocycles (2–26) in all cases were found to be less aromatic with smaller TRE and % TRE than their respective URFs (27–30). Some of the heteroatom-bridged [18]annulenes have been found to be stable compounds. Some of the di-bridged [18]annulene (11) and tri-bridged [18]annulenes (17, 19, 20, 23 and 25) have been also been found to be stable compounds by several research groups.^4,39–44^

In order to determine whether or not the [18]annulene pathway has an aromatic effect on the molecule as a whole, we decided to calculate the TRE and % TRE values of some of the possible open-chain analogues (31–59) and these model compounds are exhibited in Fig. S1 of the ESI.† These open-chain analogues (31–59) are obtained simply by removing from the parent structure the bond that links five-membered heterocyclic rings. The TRE and % TRE values for 31–59 are given in S-T1 of the ESI.† By comparing the TRE and % TRE values of compounds 31–59 with their corresponding parent compounds 2–26, we found that 31–59 still have positive TRE and % TRE values as large as those of 2–26. These results indicate that the five-membered heterocyclic ring(s) contribute significantly to the aromaticity not only in 2–26, but also in such open-chain species as 31–59.

### 3.2. Local aromaticity

Next, we explore the influence of five-membered heterocyclic rings on global and local aromaticity. The local aromaticity of the bonds of individual rings was analyzed on the basis of the BRE index, and the local aromaticity of individual rings themselves was analyzed on the basis of the CRE index.

#### 3.2.1. The BRE index

The BRE values for each of the different π-bonds in compounds 1–30 are displayed in Fig. 1. As shown in Fig. 1, all the bonds in 1–30 exhibit positive BRE values, which means all the bonds make aromatic contribution to the molecule, and they do this because the number of electrons in each ring satisfies Hückel's (4*n* + 2) π rule of aromaticity. In the five-membered heterocyclic rings, the outer C–C bonds showed relatively larger BRE values than did the inner C–X bonds. The BRE values, both of the C–C and the C–X bonds within the five-membered rings, reveal relatively larger BRE values when compared to the C–C bonds that link five-membered rings. Thus we can conclude that the five-membered rings themselves are the main source of aromatic stabilization. After incorporating five-membered rings, the BRE values of the C–C bonds that link the five-membered rings of the molecules in 2–30 decrease slightly when compared with 1. By comparing the BRE values of 5 with 11, we find that the five-membered heterocyclic rings in 5 which have two, complete five-membered units showed relatively larger BRE values than the rings in 11, which have only one complete five-membered unit. Similar behavior can be found by comparing the BRE values of 6 with 12, and those of 7 with 13. The relative magnitudes of the BRE values reflect the number of the locations of complete, five-membered units within their structures, which, in accordance with valence bond theory, is to be expected.

#### 3.2.2. The CRE index

**Fig. 3:**
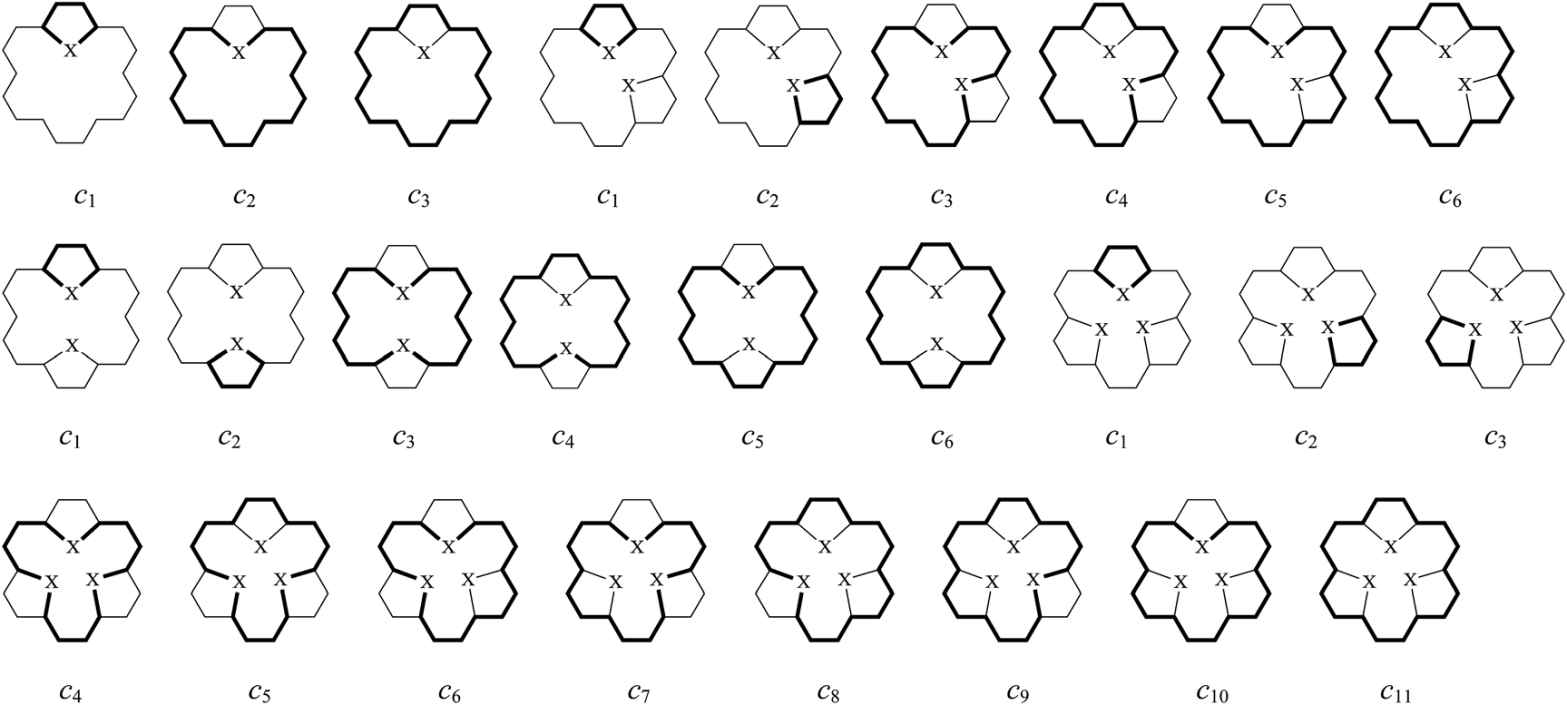
The π-electron circuits in mono-bridged (2–4), di-bridged (5–16), tri-bridged (17–26) [18]annulenes (X = O, NH and S) and the related species (27–30).

In order to further understand the individual contributions of different circuits to global aromaticity, we have also calculated the CRE values of the above compounds. As shown in Fig. 3, the π-electron ring systems of 2–4 and 27 contain 3 circuits each, which we have labeled *c*_1_, *c*_2_ and *c*_3_, respectively. The π-electron ring systems of 5–10 and 11–16 as well as that of 28 and 29 contain 6 circuits each, which we have labeled *c*_1_ to *c*_6_. The π-electron ring systems of 17–24 and 30 contain 11 circuits each, which we have labeled *c*_1_ to *c*_11_. The CRE values of compounds 1–30 are given in Table 1. As shown in that Table 1, all the circuits are aromatic with positive CRE values due to the fact that they have 6π or 18π electrons and thus satisfy the Hückel's (4*n* + 2) π rule of aromaticity. For 2, due to the fact that the electrons are mainly concentrated at the site of the highly electronegative oxygen atom, the peripheral (*c*_3_) circuit showed relatively larger positive CRE than the *c*_1_ (five-membered) and the *c*_2_ circuits. However, in 3 and 4, the peripheral (*c*_3_) circuit showed larger positive CRE values than the *c*_1_ and the *c*_2_ circuits. For all the remaining isomers (4–30), every five-membered circuit exhibited larger CRE values than the peripheral circuits and those of any other type. As the number of circuits in the molecule increases, the CRE values of the peripheral circuits decreases and the differences between the CRE values of the five-membered circuits and the peripheral circuits increases. For 27–30, all atoms in the ring are of the same electronegativity. Thus, the 6π-electrons are uniformly distributed over the five carbon atoms. However, for 2–26, the heteroatom X is more electronegative than carbon. These results in a substantial electric charge transfer from the carbon atoms to the neighboring heteroatom X. The electrons are localized at heteroatom X, which decreases the local aromatic character of this five-membered ring. Thus, the distribution of the electrons in the five-membered rings is affected by the CRE value. According to the CRE values, the increase of TRE in 2–30 is usually connected to the aromaticity of the newly produced five-membered ring(s). Thus, the enhanced TRE and % TRE values are mainly produced by the delocalization of these new five-membered 4*n* + 2 electron circuits, and as the number of five-membered heterocyclic rings in the molecule increases, the contribution of the factors which produce aromaticity also increase. According to the BRE and CRE results, we can conclude that with the exception of 2, all these compounds are primarily stabilized by their 6π-electron, five-membered systems, and the [18]annulene-like conjugation pathway is not determinative of the aromaticity of the π-system as a whole.

Magnetic resonance energy (MRE) is defined as the sum of the CREs over all circuits.^24^ The MRE values of 1–30 are given in Table 1. A high degree of correlation is found between TRE and MRE, and between % TRE and MRE, with the correlation coefficient being 0.9986 and 0.9621, respectively.

## 4. RCs and the NMR chemical shifts

**Fig. 4:**
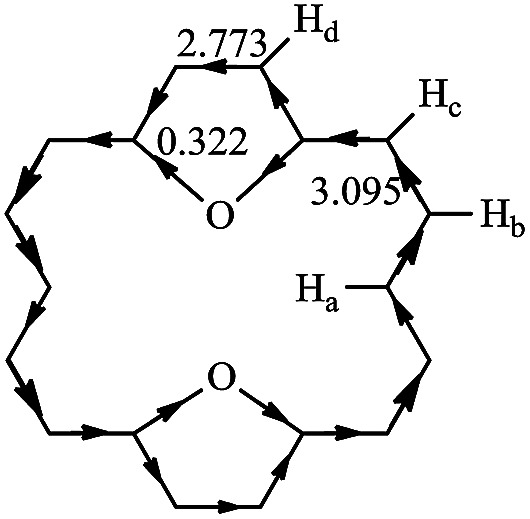
The RC for compound 11, in units of that for benzene (*I*_0_).

RC is another method to measure the stability of aromatic molecules.^45,46^ In general, the magnetically induced diatropic and paratropic ring currents are associated with aromaticity and antiaromaticity, respectively. The induced RC produces a magnetic field which flows in a direction opposite of the external field when at the center of the ring and which flows in the same direction as the external field when it is outside the ring. The NMR analysis of 1 has shown that chemical shifts of the outer protons were shifted downfield (9.28 ppm), whereas the inner protons were shifted upfield (−2.99 ppm).^3^ It is confirmed that 1 exhibits a strongly diatropic RC. In polycyclic systems, the RCs arise from the global properties of the molecule as a whole. Thus, the RC is quite difficult to analyze and evaluate. Hückel–London theory was used here to derive RC patterns for the compounds included in this study.^32–35^ The calculated CC (circuit current) values for 1–30 are given in Table 2. In general, a positive or negative CC value indicates a diatropic or a paratropic contribution respectively, to a given circuit. As can be seen in Table 2, all circuits in 1–30 exhibit positive CC values, since they are all (4*n* + 2)-electron circuits. Thus we can predict that all circuits make a diatropic contribution to the molecule. In 2–26, fairly large diatropicity arises from the peripheral circuit. Thus we can predict that the peripheral circuit contributes predominantly to the diatropicity of 2–26. However, in 27–30, fairly large diatropicity arises from the five-membered circuits. RC flow through a given ring can be calculated precisely as the sum of the corresponding CC contributions.^24^ The direction and intensity of the RCs of 11 are depicted in Fig. 4. That of the remaining compounds are given in Fig. S2.† The clockwise and counterclockwise arrows show paratropicity and diatropicity, respectively. 2–30 were predicted to sustain a strong diatropic current along the [18]annulene pathway, whereas only weak diatropic currents flow on the C–X bonds of the five-membered heterocyclic rings. The diamagnetic current induced along the macrocycle is bifurcated when it passes through each five-membered heterocyclic ring. A strong diamagnetic current is also induced at the site of each five-membered ring circuit. However, both diatropic currents partially cancel each other out on the inner C–X bonds of the five-membered heterocyclic rings, as both currents flow in opposite directions. Thus, the magnitude of the diatropic RCs flow on the outer C–C bonds is larger than the inner C–X bonds in the five-membered heterocyclic rings. The comparison of our RC values of 2–30 with 1 reveals that after the five-membered rings have been incorporated into 1, the resulting compounds 2–30 still produce strong diamagnetic RCs, but the degree of diatropicity of 2–30 is weaker than that found in 1. The analysis of very high-field ^1^H-NMR spectrum for 17, 19, 20, 23, and 25 produced results similar to the results for 1, and the existence of diatropic RCs in 17, 19, 20, 23, and 25 (similar to those in 1), was demonstrated.^4,41–44^ These results are in good accord with the diamagnetic RC values we calculated. Ogawa *et al.*^39,40^ reported the synthesis of 11. The difference between the chemical shifts of the inner protons and the outer proton of 11 was found to be larger than that same difference in 1. As shown in Fig. 4, the ^1^H-NMR spectra of 11 reveals very high field resonance of the inner protons *δ* = −5.89 (H_a_, 2H) and low field resonance of the outer protons *δ* = 10.00 (H_b_, 4H), 9.20 (H_c_, 4H), 9.13 (H_d_, 4H) (in CDCl_3_), confirming that 11 is strongly diatropic. The diatropic RC values (3.095*I*_0_) of the flow through the section attached to the H_b_ and H_c_ atoms is larger than that of the RC values (2.773*I*_0_) of the flow through the section attached to the H_d_ atom. That is to say, the diatropic RC value we calculated for the two sections within 11 is in agreement with the ^1^H NMR chemical shifts observed by Ogawa *et al.*^39,40^

**Table 2:** CC values of compounds 1–30 (all in units of that for benzene)

Species	*c*_1_	*c*_2_	*c*_3_	*c*_4_	*c*_5_	*c*_6_	*c*_7_	*c*_8_	*c*_9_	*c*_10_	*c*_11_
1	3.1427										
2	0.1634	0.5207	2.6198								
3	0.2970	1.0616	1.9838								
4	0.2298	0.8672	2.2016								
5	0.1716	0.1716	0.0825	0.4265	0.4265	2.1650					
6	0.3327	0.3327	0.3141	0.6291	0.6291	1.2101					
7	0.2534	0.2534	0.2121	0.5807	0.5807	1.5131					
8	0.1809	0.3141	0.1628	0.8571	0.3172	1.6253					
9	0.1776	0.2437	0.1333	0.7010	0.3554	1.8119					
10	0.2590	0.3260	0.2581	0.7095	0.5153	1.3545					
11	0.1581	0.1581	0.0843	0.4359	0.4359	2.2128					
12	0.2823	0.2823	0.3483	0.6956	0.6956	1.3372					
13	0.2186	0.2186	0.2271	0.6205	0.6205	1.6157					
14	0.1530	0.2895	0.1708	0.8987	0.3324	1.7033					
15	0.1549	0.2228	0.1386	0.7286	0.3691	1.8821					
16	0.2161	0.2851	0.2589	0.7097	0.5159	1.4686					
17	0.1802	0.1802	0.1802	0.0121	0.0659	0.0659	0.0659	0.3457	0.3457	0.3457	1.7728
18	0.3693	0.3693	0.3693	0.0765	0.1699	0.1699	0.1699	0.3574	0.3574	0.3574	0.7110
19	0.2778	0.2778	0.2778	0.0447	0.1328	0.1328	0.1328	0.3803	0.3803	0.3803	1.0175
20	0.1897	0.1897	0.3318	0.0232	0.1275	0.1275	0.0478	0.6845	0.2547	0.2547	1.3188
21	0.1863	0.1863	0.2582	0.0191	0.1046	0.1046	0.0542	0.5609	0.2867	0.2867	1.4767
22	0.1992	0.3507	0.3507	0.0428	0.2415	0.0913	0.0913	0.4970	0.4970	0.1858	0.9727
23	0.1923	0.2680	0.2680	0.0293	0.1639	0.0857	0.0857	0.4622	0.4622	0.2372	1.2275
24	0.2890	0.3625	0.3625	0.0637	0.1959	0.1404	0.1404	0.4073	0.4073	0.2939	0.8032
25	0.2834	0.2834	0.3557	0.0530	0.1615	0.1615	0.1157	0.4630	0.3348	0.3348	0.9051
26	0.1958	0.2736	0.3438	0.0354	0.1989	0.1043	0.0751	0.5636	0.4080	0.2102	1.0939
27	0.3082	2.0991	0.2751								
28	0.4475	0.4475	0.8484	0.2527	0.2527	0.0233					
29	0.2963	0.2963	1.4451	0.3941	0.3941	0.0444					
30	0.5497	0.5497	0.5497	0.1540	0.1032	0.1032	0.1032	0.0372	0.0372	0.0372	0.0014

## 5. Conclusions

In this study, we have investigated the aromaticity and RCs of mono-bridged, di-bridged, and tri-bridged [18]annulenes and designed new compounds. Our important findings are summarized below:

(1) The incorporation of one, two, or three, five-membered heterocyclic rings into the [18]annulene produces new aromatic rings and causes change in the main conjugation pathways of these compounds. The production of new aromatic five-membered heterocyclic rings results in the overall stabilization of the system and tends to increase the TRE and % TRE indices.

(2) All compounds studied were found to be aromatic and diamagnetic. For 2–26, aromaticity mainly arose from the 6π-electron five-membered circuits. However, diatropicity arose primarily from 18π-electron peripheral circuits. Because the main source of the aromaticity and of the RCs was not the same for these compounds, the degree of local aromaticity of these compounds cannot be accurately determined from the RCs.

(3) Our RC results show that a strong diamagnetic current flows around the entire molecular perimeter and never passes through heteroatoms in the five-membered ring(s). For those compounds which were synthesized, our RC results are consistent with the observed ^1^H-NMR chemical shifts.

## Conflicts of interest

There are no conflicts to declare.

## Supplementary Material

RA-009-C9RA04193A-s001
