# Supplementary material for: A study on the aromatic conjugation pathways and the ring currents of bridged [18]annulenes
Source: RSC Adv. 2019 Aug 14;9(44):25351–7. doi: 10.1039/c9ra04193a (PMC9070026; doi:10.1039/c9ra04193a)
Supplement: RA-009-C9RA04193A-s001 [file RA-009-C9RA04193A-s001.pdf]

Electronic Supplementary Material (ESI) for RSC Advances.
This journal is © The Royal Society of Chemistry 2019

Electronic Supplementary Information (ESI) for

A Study on the Aromatic Conjugation Pathways and the Ring Currents of Bridged [18]Annulenes

Qimangul Tohti and Ablikim Kerim

The College of Chemistry and Chemical Engineering, Xinjiang University, Urumqi 830046, China
Email: ablikim.kerim@163.com

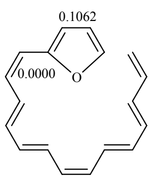

31

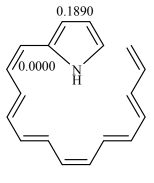

32

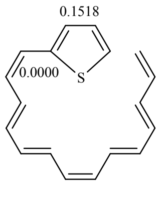

33

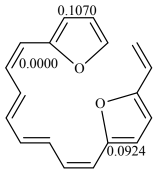

34

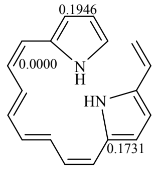

35

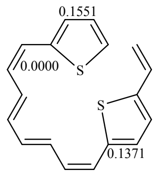

36

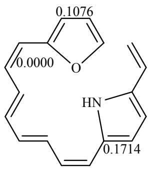

37

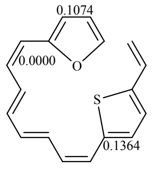

38

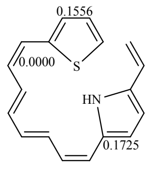

39

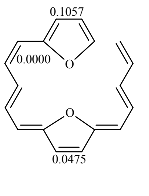

40

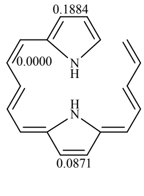

41

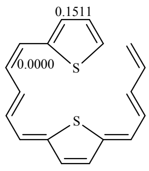

42

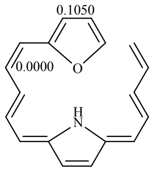

43

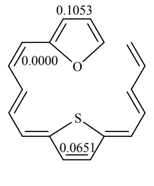

44

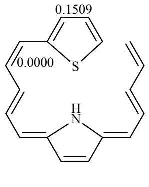

45

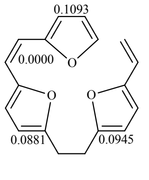

46

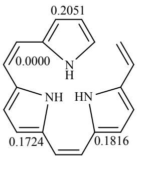

47

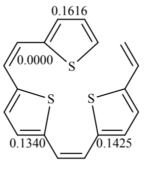

48

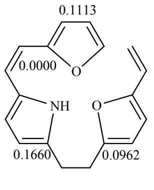

49

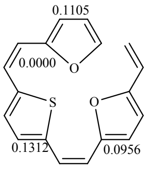

50

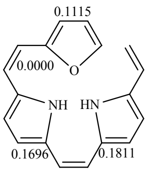

51

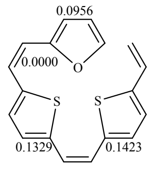

52

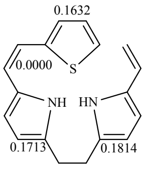

53

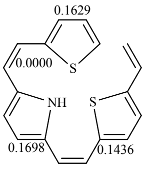

54

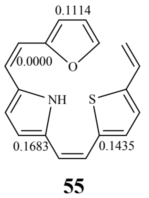

55

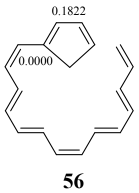

56

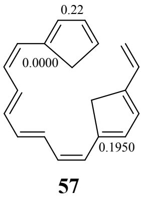

57

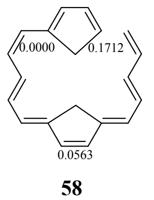

58

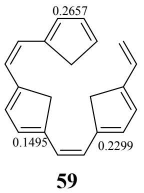

59

S-Fig1. Reference molecules of **2-30**. BREs in units of |β| for all π-bonds.

S-Table 1. The TRE and %TRE values of compounds **31-59**.

Species	TRE	%TRE	Species	TRE	%TRE
**31**	0.106	0.398	**46**	0.289	0.810
**32**	0.189	0.736	**47**	0.550	1.694
**33**	0.152	0.609	**48**	0.431	1.425
**34**	0.199	0.639	**49**	0.368	1.065
**35**	0.367	1.263	**50**	0.333	0.984
**36**	0.292	1.057	**51**	0.455	1.358
**37**	0.278	0.925	**52**	0.380	1.187
**38**	0.243	0.828	**53**	0.508	1.600
**39**	0.328	1.156	**54**	0.468	1.512
**40**	0.154	0.494	**55**	0.417	1.271
**41**	0.281	0.965	**56**	0.182	0.761
**42**	0.220	0.796	**57**	0.424	1.662
**43**	0.193	0.640	**58**	0.278	1.095
**44**	0.172	0.585	**59**	0.717	2.662
**45**	0.241	0.852			

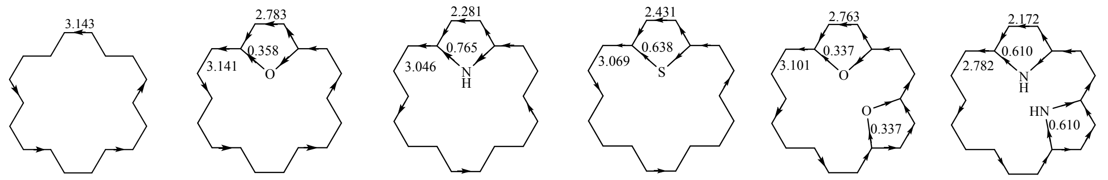

1 **2** **3** **4** **5** **6**

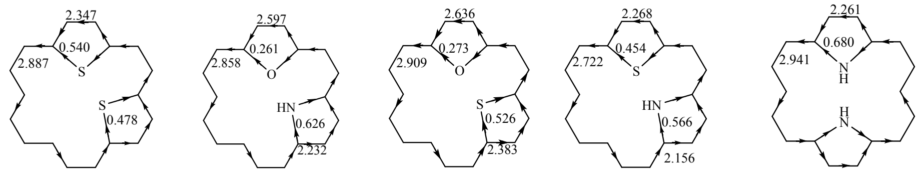

7 **8** **9** **10** **12**

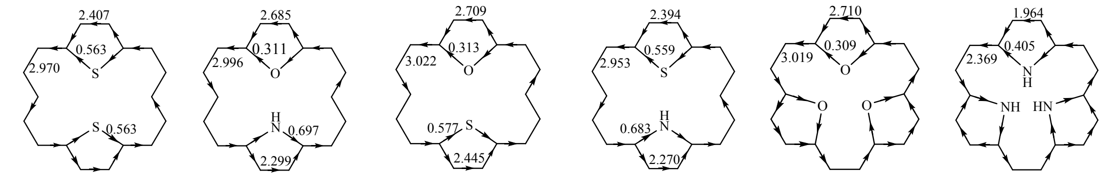

13 **14** **15** **16** **17** **18**

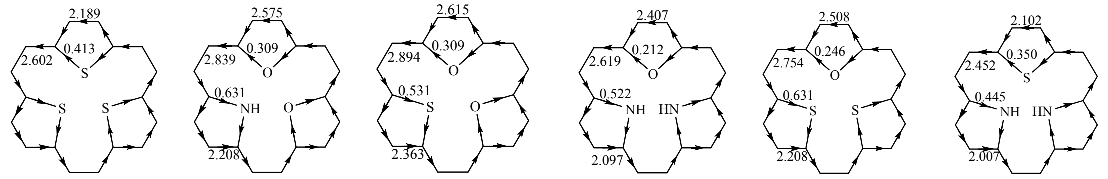

19 **20** **21** **22** **23** **24**

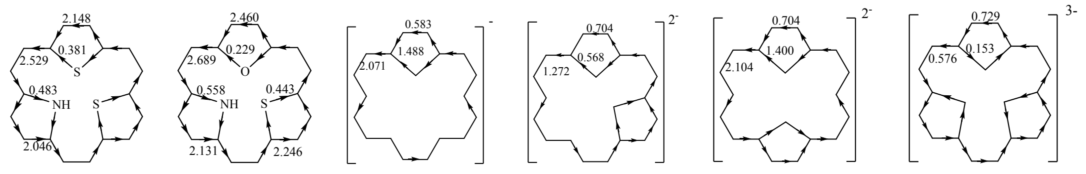

25 **26** **27** **28** **29** **30**

S-Fig. 2. The RCs of the remaining compounds, all in units of that for benzene (I_0)
